# Value of adding the apparent diffusion coefficient to capsular contact for the prediction of extracapsular extension in prostate cancer

**DOI:** 10.1590/0100-3984.2019.0123

**Published:** 2020

**Authors:** Antonio Cordeiro da Silva Filho, Tamara Oliveira Rocha, Jorge Elias Jr, Marcus Vinicius de Castro Barros, Alfredo Ribeiro Silva, Rodolfo Borges dos Reis, Valdair Francisco Muglia

**Affiliations:** 1 Faculdade de Medicina de Ribeirão Preto da Universidade de São Paulo (FMRP-USP), Ribeirão Preto, SP, Brazil.

**Keywords:** Prostatic neoplasms, Diffusion magnetic resonance imaging, Magnetic resonance imaging, Neoplasm staging, Neoplasia da próstata, Difusão, Ressonância magnética, Estadiamento de neoplasias

## Abstract

**Objective:**

To determine whether evaluating the mean apparent diffusion coefficient (ADC) together with capsular contact (CC) adds value in the prediction of microscopic extracapsular extension (ECE) of prostate cancer.

**Materials and Methods:**

Between January 2012 and December 2016, 383 patients underwent multiparametric magnetic resonance imaging (mpMRI) of the prostate. A total of 67 patients were selected for inclusion. Two radiologists (observers 1 and 2), working independently, performed qualitative and quantitative analyses of ECE, macroscopic ECE, and microscopic ECE. A third radiologist assessed the correlation with the clinical data, and two experienced pathologists reviewed all histopathological findings.

**Results:**

Among the 67 patients, mpMRI showed lesions that were confined to the capsule in 44 (66.7%), had microscopic ECE in 12 (17.9%), and had macroscopic ECE in 11 (16.4%). There were no significant differences, in terms of the diagnostic accuracy, as measured by determining the area under the curve (AUC), of CC on T2-weighted images (CCT2), CC on diffusion-weighted imaging (CCDWI), and the mean ADC for the prediction of microscopic ECE, between observer 1 (AUC of 0.728, 0.691, and 0.675, respectively) and observer 2 (AUC of 0.782, 0.821, and 0.799, respectively). Combining the mean ADC with the CCT2 or CCDWI did not improve the diagnostic accuracy for either observer. There was substantial interobserver agreement for the qualitative evaluation of ECE, as demonstrated by the kappa statistic, which was 0.77 (0.66-0.87). The diagnostic accuracy (AUC) of the qualitative assessment for predicting microscopic ECE was 0.745 for observer 1 and 0.804 for observer 2, and the difference was less than significant. In a multivariate analysis, none of clinical or imaging parameters were found to be associated with ECE.

**Conclusion:**

For the detection of microscopic ECE on mpMRI, CC appears to have good diagnostic accuracy, especially if the observer has considerable experience. Adding the mean ADC to the CCT2 or CCDWI does not seem to provide any significant improvement in that diagnostic accuracy.

## Abstract (pt)

**Objetivo:**

Avaliar se o coeficiente de difusão aparente (*apparent diffusion coefficient* - ADC) médio tem valor incremental ao contato capsular (CC) na predição da extensão extracapsular (EEC) do câncer de próstata.

**Materiais e Métodos:**

De janeiro de 2012 a dezembro de 2016, 383 pacientes realizaram ressonância magnética multiparamétrica de próstata. Após os critérios de inclusão e exclusão, 67 pacientes foram selecionados para avaliação qualitativa e quantitativa, por dois radiologistas independentes, da EEC, EEC grosseira e EEC microscópica. Um terceiro observador coletou dados clínicos e dois patologistas experientes revisaram os achados histopatológicos.

**Resultados:**

Dos 67 pacientes selecionados, 44 apresentaram lesões restritas à cápsula (66,7%), 12 com EEC microscópica (17,9%) e 11 com EEC grosseira (16,4%). Não houve diferença significativa entre a acurácia diagnóstica, medida pela área sob a curva, entre o CC na ponderação T2 (CCT2), CC-difusão e ADC para predição da EEC microscópica para ambos os observadores (0,728, 0,691 e 0,675, respectivamente, para o observador 1, e 0,782, 0,821 e 0,799, respectivamente, para o observador 2). A associação dos valores médios do ADC ao CCT2 e ao CC-difusão não promoveu melhora da acurácia diagnóstica. A concordância interobservador para a avaliação qualitativa da EEC mostrou coeficiente kappa de 0,77 (0,66-0,87), inferindo concordância substancial. A acurácia da avaliação qualitativa para EEC microscópica foi de 0,745 e 0,804 para os observadores 1 e 2, respectivamente, diferença não significativa. Na análise multivariada, nenhum parâmetro clínico ou de imagem foi associado a EEC.

**Conclusão:**

O CC mostrou boa acurácia diagnóstica para a detecção de EEC microscópica, especialmente para o observador mais experiente. A inclusão dos valores médios de ADC não melhorou a acurácia do CC para predição de EEC microscópica.

## INTRODUCTION

Prostate adenocarcinoma is the second leading type of malignant neoplasm in men, surpassed only by nonmelanoma skin cancer in terms of incidence and by lung cancer in terms of mortality^(1)^. Radical prostatectomy is the treatment of choice for prostate cancer, providing excellent disease-specific survival for localized cancers^(2)^. Although the procedure is performed for curative purposes, it is also aimed at preserving the neurovascular bundle, preventing erectile dysfunction and urinary incontinence (the so-called trifecta outcomes of urinary continence, sexual potency, and cancer control), because these possible postoperative complications have a negative impact on patient quality of life^(3)^.

The extracapsular extension (ECE) of prostate neoplasms is an important prognostic factor because it is associated with an increased risk of postoperative biochemical recurrence; it also has important therapeutic implications because, when it is present, a more radical surgical resection is indicated to reduce the chance of positive surgical margins^(4)^. However, when the ECE nears the neurovascular bundle, there is an increased risk of injury to that structure^(4,5)^. Therefore, for better therapeutic planning and a more well-informed decision-making process aimed at cancer control with preservation of erectile function and urinary continence, it is extremely important to carry out a preoperative assessment of the risk of ECE.

Multiparametric magnetic resonance imaging (mp MRI) of the prostate has become an important tool, not only for the detection of prostate cancer but also for its staging^(6)^. Imaging-based staging usually implies a morphological evaluation on T2-weighted sequences. In addition to showing the capsule, such sequences can show gross extensions as well as suspicious findings that can be indicative of microscopic ECE, such as in cases in which there is considerable contact between the neoplasm and the prostatic capsule^(7)^. Since the advent of new MRI resources, such as the incorporation of functional methods-diffusion weighted imaging (DWI), dynamic contrast-enhanced imaging, and spectroscopy-there have been various studies evaluating their role in predicting microscopic ECE^(8-12)^. More recently, studies focusing on ECE have started to include the analysis of DWI, an important component of mpMRI scans^(13)^. Rosenkrantz et al. reported cutoff values of capsular contact for predicting any ECE, which were 7 mm on the apparent diffusion coefficient (ADC) map and 6 mm on T2-weighted images. The cutoff values of capsular contact for predicting nonfocal ECE were 7.0 mm on the ADC map and 10.0 mm on T2-weighted images. According to the authors, those cutoff values can yield good results for predicting microscopic ECE^(14)^, with only a slight loss of specificity. However, in most other studies that have addressed this topic, the reported cutoff values of capsular contact ranged from 12 mm to 18 mm for T2-weighted images^(8,9,13)^.

Because the ADC map and its derived parameters, obtained from DWI, have been considered potential biomarkers of prostate cancer aggressiveness, having an inversely proportional correlation with the Gleason score, some studies have analyzed the possibility that the ADC can be an independent predictor of ECE^(15,16)^ or that it can add value to capsular contact measurement. Until recently, the mean ADC value was considered useful in predicting ECE only when the T2-weighted images were inconclusive^(17)^. Version 1 of the American College of Radiology Prostate Imaging Reporting and Data System (PI-RADS) included a risk stratification for the prediction of ECE in lesions with a final PI-RADS score > 3^(18)^, although that suggestion was omitted from version 2.0^(19)^. There is no consensus in the literature regarding the optimal capsular contact threshold for predicting microscopic ECE or regarding the best sequences to be used and the role of DWI in evaluating capsular contact^(9-13,15,17)^.

The objective of the present study was to evaluate whether DWI, and more specifically the calculation of the mean ADC value, can improve the specificity and sensitivity of microscopic ECE detection in prostate cancer, based on the degree of capsular contact, and to determine the best sequence to measure capsular contact-T2-weighted images or DWI (ADC mapping)-using the histopathological results obtained after radical prostatectomy as a reference standard. A secondary objective was to assess the reproducibility of the definition of ECE based on the parameters described above, between observers with different levels of experience in the field.

## MATERIALS AND METHODS

The study was approved by the research ethics committee of our institution. Because of the retrospective nature of the study, the requirement for written informed consent was waived.

We searched the hospital database of our institution to identify patients who had undergone mpMRI of the prostate followed by radical prostatectomy between January 2012 and December 2016. We included consecutive patients with prostate cancer who had undergone preoperative evaluation including MRI of the prostate, followed by radical prostatectomy within a three-month period. Patients in whom the protocol for mpMRI of the prostate was not completed were excluded, as were those in whom the histopathological study was incomplete and those who had previously undergone treatment for prostate cancer.

Of a total of 383 patients initially selected, 316 were excluded: because they had undergone mpMRI but did not undergo surgery (n = 98); because they had previously undergone treatment (surgery, radiotherapy, or hormone therapy) for prostate cancer (n = 26); because the recommended mpMRI protocol was not followed (n = 30); because the images obtained were considered inappropriate because of magnetic susceptibility artifacts (n = 38); because the interval between the MRI examination and the surgery was longer than three months (n = 112); or because the quality of the surgical specimen was unsatisfactory (n = 12). Therefore, the final sample comprised 67 patients.

The clinical data were analyzed by a radiologist from our institution different from the ones who read the MRI scans. The following parameters were considered: age; serum level of prostate specific antigen (PSA); clinical staging; biopsy date; mpMRI date; surgery date; interval between mpMRI and surgery; and the Gleason score/International Society of Urological Pathology (ISUP) score for prostatectomy.

The MRI scans of the prostate were acquired in a 16-channel 1.5-T scanner (Achieva; Philips Medical Systems, Best, Netherlands). The protocol used is detailed in Table 1. We employed OsiriX viewer software, version 2.6 (Pixmeo Sàrl, Geneva, Switzerland).

**Table 1:** Acquisition parameters for the MRI protocol.

Plane and weighting	Sequence	TR/TE (ms)	Flip angle (°)	Slice thickness (mm)	Field of view (cm)	Matrix	Maximum b value
Axial T2	TSE	3060/100	90	3	150 × 150	232 × 184	—
Coronal T2	TSE	2444/120	90	3	150 × 150	248 × 198	—
Sagittal T2	TSE	3770/120	90	3	260 × 260	360 × 275	—
Axial DWI	SE EPI	1561/71	90	5	304 × 375	152 × 152	1000/1400*
Axial perfusion	THRIVE	43.865	10	4	297 × 345	172 × 172	—
Axial T1	TSE	443/15	90	3	180 × 180	180 × 143	—

TR/TE, repetition time/echo time; TSE, turbo spin-echo; SE, spin-echo; EPI, echo-planar imaging; THRIVE, T1-weighted high resolution isotropic volume examination.

* Value of b expressed in s/mm^2^.

### Image interpretation

The images were read by two radiologists working independently (ACSF-observer 1, with four years of experience in radiology and two years of experience in MRI of the prostate; and VFM-observer 2, with 14 years of experience in prostate imaging).

For the qualitative assessment, the following parameters were considered: location of the lesion, described in accordance with the recommendations of the PI-RADS, version 2.1^(20)^; measurement of the longest axis of the lesion; and the PI-RADS classification for each lesion (on DWI, on T2-weighted images, and overall). The ECE investigation started with the qualitative assessment of the T2-weighted images, and the findings were classified as follows: 1 - no capsular contact; 2 - capsular contact but no bulging; 3 - capsular contact with bulging; 4 - macroscopic ECE (visible, > 1.0 mm). For the purpose of calculating the diagnostic accuracy of the qualitative assessment, findings 1 and 2 were considered negative, and findings 3 and 4 were considered positive for ECE (microscopic and macroscopic, respectively).

**Figure 1:**
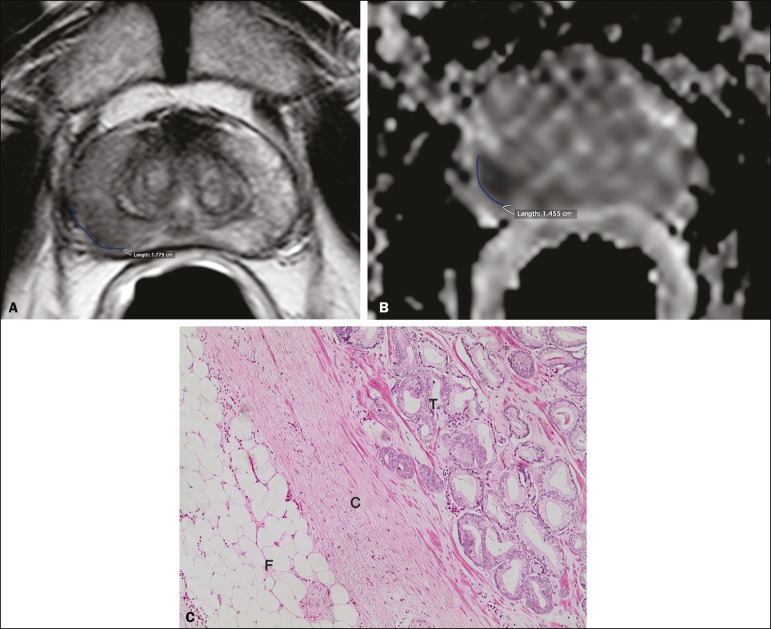
A 63-year-old male patient with a serum PSA of 7.51 ng/dL. Prostate cancer with a Gleason score of 7 (4+3, 6 positive fragments out of 12) and a clinical stage of 2a. **A:** Axial T2-weighted image showing the absence of macroscopic ECE, the extent of capsular contact being determined to be 21.0 mm by observer 1 and 17.8 mm by observer 2 (blue line). **B:** ADC map showing the extent of capsular contact, which was determined to be 17.9 mm by observer 1 and 14.5 mm by observer 2 (blue line). **C:** Histological section; hematoxylin-eosin staining shows the tumor tissue (T), a preserved capsule (C), and periprostatic fat (F).

For the quantitative analysis, the extent of capsular contact was measured, in mm, on T2-weighted images (Figure 1A) and on the ADC map (Figure 1B). The mean ADC (10^−3^ mm^2^/s) was calculated from the measurements obtained in the images in which the lesion was visualized, using regions of interest manually marked by each of the observers, working independently.

### Histopathologic analysis

The histopathological analysis was conducted by two pathologists from our institution, with 20 and 8 years of experience, respectively, in urological specimen pathology. The standard procedure for analyzing radical prostatectomy specimens at our laboratory includes slicing the specimen at every 5.0 mm in the transversal plane, and the histological tests are then performed on those sections. Following recommendations in the literature^(21)^, we defined three possible results for all tumor foci in contact with the prostatic capsule: absence of ECE; microscopic ECE, defined as the presence of tumor tissue beyond the capsule that does not exceed a wide (< 0.5 mm) field of view; and macroscopic ECE (visible on MRI), when the tumor tissue exceeds the limit defined for microscopic ECE. A third radiologist then made the correlation between the sites defined by the other two observers in the image interpretation and the pathology study results, using the map recommended in the PI-RADS, version 2.1^(20)^.

### Statistical analysis

The statistical analysis was performed with Stata software, version 15.0 (Stata Corp., College Station, TX, USA). Continuous variables are expressed as mean and standard deviation (and/or 95% confidence interval) or as median (and range)

The diagnostic accuracy for identifying ECE was determined by calculating the area under the (receiver operating characteristic) curve (AUC). The method proposed by DeLong et al^(22)^ was employed in order to identify the best cutoff values for classifying ECE (mean ADC and extent of capsular contact on T2-weighted images and DWI). To analyze the interobserver agreement of the qualitative assessment of T2-weighted images, PI-RADS DWI, PI-RADS T2-weighted images, and overall PI-RADS, we used simple Cohen’s Kappa^(23)^. Logistic regression and odds ratios were used in order to assess the possibility of the following variables being predictors of ECE: serum PSA level, Gleason score, clinical stage, percentage of tumor-positive fragments on biopsy, and qualitative assessment of T2-weighted images. In all comparisons, values of *p* < 0.05 were considered significant.

## RESULTS

The mean age of the patients in our study sample was 63.3 ± 7.1 years (46-76 years). The mean serum PSA level was 10.9 ± 5.1 ng/dL, and the median serum PSA level was 7.9 ng/dL (range, 3.4-41.4 ng/dL). The values for each group are displayed in Table 2.

**Table 2:** Demographic, clinical, and histopathological characteristics.

Characteristic	No ECE (n = 44)	Microscopic ECE (n = 12)	Macroscopic ECE(n = 11)	*P*-value
Age (years)	63.8 ± 7.1	61.8 ± 9.5	64.4 ± 6.9	0.75
Serum PSA (ng/dL)	9.5 ± 5.86	9.83 ± 6.86	19.9 ± 11.98	0
Lesion size (mm)	13.3 ± 4.6	17.6 ± 6.4	22.4 ± 13.2	0
Clinical stage				0.0004
T1	18 (40.9)	1 (8.3)	0	
T2a	11 (25.0)	4 (33.3)	0	
T2b	9 (20.4)	4 (33.3)	1 (9.1)	
T2c	6 (13.7)	1 (8.3)	2 (18.2)	
T3a/b	0	2 (16.7)	8 (66.7)	
Gleason score (ISUP grade)				0.0001
3+3 (1)	13 (29.5)	0	0	
3+4 (2)	18 (40.9)	5 (41.7)	2 (18.1)	
4+3 (3)	9 (20.5)	6 (50.0)	3 (27.3)	
4+4, 3+5, 5+3 (4)	3 (6.8)	0	3 (27.3)	
4+5, 5+4, 5+5 (5)	1 (2.3)	1 (8.3)	3 (27.3)	
Positive fragments on biopsy (%)	37.7 ± 24.6	49.3 ± 21.2	57.5 ± 23.7	0.02
PI-RADS classification				0.0001
2	2 (4.6)	0	0	
3	10 (22.7)	0	0	
4	23 (52.3)	5 (41.7)	0	
5	9 (20.4)	7 (58.3)	11 (100)	
Mean ADC (10^–3^ mm^2^/s)*	1.241 ± 0.26	1.13 ± 0.34	0.96 ± 0.18	0.01
CCT2 (mm)*	9.0 ± 8.1 (0–21)	18.3 ± 9.0 (8–37)	24.0 ± 14.4 (13–65)	0.00001
CCDWI (mm)*	10.2 ± 8.6 (0–24)	22.7 ± 11.1 (7–41)	25.0 ± 15.5 (11–65)	0.00001

* Mean of the values measured by both observers. The values measured by each observer can be found in the text.

The mean interval between MRI and surgery was 42.2 days (1-90 days). In the analysis of the images, observer 1 found the mean tumor size to be 1.7 cm (0.8-6.1 cm), whereas observer 2 found it to be 1.6 cm (0.7-5.9 cm).

According to the histopathological results, 64.2% of the patients had a total Gleason score of 7-38.8% with a Gleason score of 3+4 (ISUP grade of 2) and 25.4% with a Gleason score of 4+3 (ISUP grade of 3)-which is indicative of an intermediate risk for aggressive cancer, and 16.5% had a total Gleason score of 8 or 9 (ISUP grade of 4 or 5), which is indicative of high-grade cancer. The distribution of clinical, demographic, and histopathologic data is shown in Table 2.

When measured on T2-weighted images, the mean extent of contact between the neoplasm and the capsule was 14.6 ± 11.7 mm (0-55.0 mm) for observer 1 and 13.1 ± 11.2 mm (0-65.0 mm) for observer 2. On DWI, using the ADC map, the mean extent of contact was 16.9 ±12.7 mm (0-57.0 mm) for observer 1 and 14.9 ± 12.2 mm (0-63.0 mm) for observer 2.

The mean ADC, calculated from the values obtained by both radiologists, was 1.241 ± 0.26 × 10^-3^ mm/s^2^ for the group with no ECE, 1.13 ± 0.34 × 10^-3^ mm/s^2^ for the group with microscopic ECE, and 0.96 ± 0.18 × 10^-3^ mm/s^2^ for the group with macroscopic ECE (Table 2), the difference among the three groups was statistically significant (*p* = 0.01). The histological evaluation of the surgical specimen margins (Table 2) showed that 65.7% of patients had no ECE, 17.9% (n = 12) had microscopic ECE, and 16.4% (n = 11) had macroscopic ECE. The qualitative assessment made by the two observers using the T2-weighted images acquired with mpMRI showed that there was a predominance of condition 2 (lesion slightly touching the capsule), seen by observer 1 in 46.3% of the patients and by observer 2 in 44.8% of the patients.

There was substantial interobserver agreement for the qualitative assessment of the capsule on T2-weighted images, as demonstrated by a kappa value of 0.77 (95% CI: 0.66-0.87). The diagnostic accuracy for the prediction of microscopic ECE was 0.745 (95% CI: 0.603-0.856) for observer 1, compared with 0.804 (95% CI: 0.675-0.897) for observer 2, and the difference was not significant (*p* = 0.92). For the detection of microscopic and macroscopic ECE (Table 3), the diagnostic accuracy was 0.716 (95% CI: 0.593-0.820) for observer 1 and 0.761 (95% CI: 0.641-0.857) for observer 2, another difference that was not significant (*p* = 0.97). There was also substantial interobserver agreement for the final PI-RADS classification, with a kappa statistic of 0.61 (95% CI: 0.47-0.73).

**Table 3:** Diagnostic accuracy of the qualitative assessment of microscopic ECE only and of all cases of ECE (microscopic + macroscopic)

Finding	Sensitivity	Specificity	PPV	NPV	Accuracy
Microscopic ECE					
Observer 1	0.583 (0.276–0.848)	0.794 (0.635–0.907)	0.466 (0.286–0.656)	0.861 (0.757–0.925)	0.745 (0.603–0.856)
Observer 2	0.500 (0.218–0.789)	0.886 (0.754–0.962)	0.545 (0.306–0.765)	0.867 (0.785–0.920)	0.803 (0.675–0.897)
Microscopic + macroscopic ECE					
Observer 1	0.522 (0.306–0.732)	0.818 (0.673–0.918)	0.600 (0.417–0.758)	0.766 (0.676–0.839)	0.716 (0.593–0.820)
Observer 2	0.500 (0.736–0.930)	0.889 (0.759–0.963)	0.687(0.466–0.847)	0.784 (0.703–0.848)	0.761 (0.641–0.857)

PPV, predictive positive value; NPV, negative predictive value.

**Figure 2:**
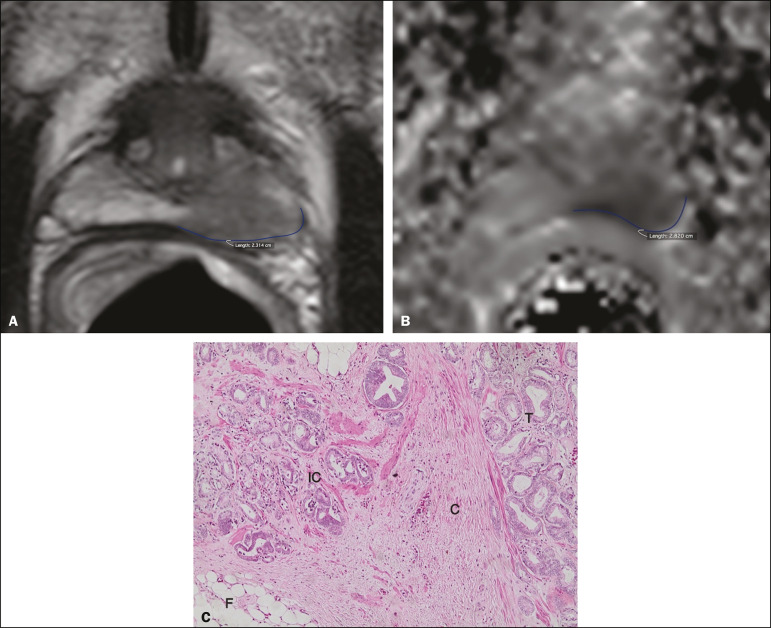
A 60-year-old male patient, serum PSA = 25.6 ng/dL. Prostate cancer with a Gleason score of 7 (4+3, 7 positive fragments out of 12) and a clinical stage of 2b. **A:** Axial T2-weighted image showing slight bulging of the capsule but no conclusive signs of macroscopic ECE, the extent of capsular contact being determined to be 22.1 mm by observer 1 and 23.1 mm by observer 2 (blue line). **B:** ADC map showing the extent of capsular contact, which was determined to be of 23.1 mm by observer 1 and 28.2 mm by observer 2 (blue line). **C:** Histological section; hematoxylin-eosin staining shows the tumor (T) extending beyond the capsule (C) with an intracapsular component (IC) and tumor tissue approaching the periprostatic fat (F).

For predicting microscopic ECE, there was no significant difference between capsular contact on T2-weighted images (CCT2), capsular contact on DWI (CCDWI), and mean ADC, in terms of the AUC values, which were 0.728, 0.691, 0.675, respectively, for observer 1 and 0.782, 0.821, and 0.799, respectively, for observer 2 (Figure 2). When the mean ADC values were combined with the extent of capsular contact, the diagnostic accuracy was 0.714 for CCT2+ADC and 0.678 for CCDWI+ADC for observer 1, compared with 0.869 for CCT2+ADC and 0.870 for CCDWI+ADC for observer 2 (Table 4). The difference between the highest values of ADC+extent of capsular contact was not significant in any of the cases, with any of the parameters isolated, for either observer. The optimal cutoff value, which would maximize the AUC, was 18.0 mm for CCT2 for both observers, whereas the optimal cutoff value for CCDWI was 14.0 mm for observer 1 and 15.0 mm for observer 2.

**Table 4:** Diagnostic accuracy based on the AUC for capsular contact as measured by both observers on T2-weighted images and DWI.*

Finding	CCT2	CCDWI	ADC	ADC+CCT2	ADC+CCDWI
Microscopic ECE					
Observer 1	0.728 (0.567–0.889)	0.691 (0.523–0.889)	0.675 (0.480–0.871)	0.714 (0.537–0.890)	0.678 (0.505–0.804)
Observer 2	0.782 (0.644–0.919)	0.821 (0.687–0.950)	0.799 (0.640–0.954)	0.869 (0.780–0.959)	0.870 (0.778–0.961)
Microscopic + macroscopic ECE					
Observer 1	0.758 (0.642–0.875)	0.743 (0.623–0.863)	0.778 (0.651–0.906)	0.757 (0.643–0.878)	0.741 (0.615–0.864)
Observer 2	0.833 (0.736–0.930)	0.832 (0.730–0.934)	0.855 (0.757–0.954)	0.871 (0.782–0.960)	0.849 (0.752–0.945)

* Results shown as AUC (95% confidence interval).

For the detection of microscopic and macroscopic ECE combined, the AUC for CCT2, CCDWI, and mean ADC were 0.758, 0.743, and 0.778, respectively, for observer 1, and 0.833, 0.832, and 0.855, respectively, for observer 2. When the mean ADC values were added to capsular contact, the accuracy for detecting microscopic and macroscopic ECE was 0.757 for CCT2+ADC and 0.743 for CCDWI+ADC for observer 1 and 0.871 for CCT2+ADC and 0.849 for CCDWI+ADC for observer 2 (Table 4). Once again, there was no significant difference between the highest values of ADC+extent of capsular contact with any of the parameters isolated, for either observer.

When logistic regression was used in order to determine which variables were independent predictors of microscopic ECE, we found that serum PSA level, lesion size, Gleason score/ISUP grade, percentage of tumor-positive fragments on biopsy, CCT2, CCDWI, and mean ADC were predictors only in the univariate analysis. In the multivariate analysis, the mean ADC, in isolation or in combination with any other variable, did not correlate significantly with the occurrence of microscopic ECE.

## DISCUSSION

The use of mpMRI has become more widespread in prostate cancer, not only in the diagnostic workup but also for other purposes, such as staging. In the present study, combining the mean ADC values of the lesions with the morphological variables did not seem to improve diagnostic accuracy for the identification of ECE. However, the extent of tumor-capsule contact proved to be a better predictor of microscopic ECE when measured on the ADC map (DWI) than when measured on T2-weighted images.

Because more refined surgical techniques require better therapeutic planning based on more accurate preoperative information, the use of mpMRI for prostate cancer staging has grown in recent years. Tumor-positive surgical margins have been associated with biochemical recurrence^(24)^, shorter disease-free (recurrence-free) survival, and the need for rescue therapies^(25)^. Although it is known that prostate cancer outcomes are highly dependent on the skills and experience of the attending urologist, thorough surgical planning also seems to be relevant^(26,27)^.

In the 1990s, Smith et al.^(28)^ warned about the limitations of digital rectal examination and transrectal ultrasound for detecting ECE. Thereafter, the so-called nomograms, which are tools for predicting ECE on the basis of clinical and biochemical data, were developed. Partin et al.^(29)^ described a nomogram for the prediction of ECE that took into account factors such as serum PSA level, clinical staging, and the Gleason score on biopsy. However, their nomogram did not predict which side would be affected by the ECE, reducing its importance as a surgical planning tool. Steuber et al.^(30)^ and Sayid et al.^(31)^ developed another nomogram, also based on clinical and biochemical data, that predicted the side of the ECE (the side-specific nomogram).

More recently, other prediction tools have combined mpMRI findings with clinical data, aiming to improve the side-specific detection of ECE. The nomogram developed by Giganti et al.^(32)^ used imaging findings in combination with clinical and biochemical parameters, thus achieving better results than did nomograms that used only biochemical data. Moreover, it is also known that tools combining imaging data with clinical and biochemical data do not outperform those that use imaging data alone^(33)^. Although we did not compare nomograms that use only imaging data with nomograms that use clinical and biochemical data, we found that the values obtained by imaging-based staging were similar to those obtained with the Giganti et al.^(32)^ nomogram.

The well-known spatial resolution limitations of MRI to identify microscopic extensions is, to a large extent, offset by the possibility of evaluating tumor-capsule contact, an imaging parameter that has been used for this purpose in transrectal ultrasounds since the 1990s, as described by Shinohara et al.^(34)^. Various studies have used tumor-capsule contact as an indirect parameter for identifying microscopic ECE^(8-13,35-36)^. The optimal cutoff to achieve good sensitivity without negatively affecting specificity has been found to be close to the 18.0 mm suggested by Shinorara et al.^(34)^. In our study, DWI was superior to T2-weighted images for the detection of microscopic ECE. The cutoff values we obtained were 15.0 mm for T2-weighted images and 14.0 mm for DWI (ADC mapping), the latter showing better results than the former for both observers. Our results are in agreement with those of other studies in the literature, such as those conducted by Shinohara et al.^(34)^ and Saylu et al.^(36)^, and in disagreement with the most recent values found by Rosenkrantz et al.^(14)^, who obtained cutoff values of 7.0 mm and 6.0 mm for an ADC map and T2-weighted images, respectively.

The difference in the values we found in our study and those found by Rosenkrantz et al.^(14)^ could be related to the MRI technique employed and the criteria used in order to define microscopic ECE, given that some pathologists define it as tumor growing beyond the capsule, whereas others say it depends on whether or not the tumor has infiltrated the periprostatic fat^(37)^.

A recent study conducted by Kim et al.^(13)^ found that the measurement taken on an ADC map is a significant independent variable for predicting microscopic ECE in low-grade tumors, indicating that DWI can also be used as an important prostate cancer staging tool. Our study confirms that the mean ADC value can be an independent predictor of microscopic ECE. In addition, the extent of capsular contact, as measured on the ADC map, proved to be important for diagnosing ECE and superior to that measured on T2-weighted images. It is important to note that, despite the different techniques, our results were similar to those found by Kim et al.^(13)^ The MRI scans in our study were acquired in a 1.5-T scanner with an endorectal coil, the ADC value representing the mean of three measurements of ROIs of equal size, whereas Kim et al.^(13)^ used a 3.0-T MRI scanner without an endorectal coil and the mean of two measurements of the same region of interest. Therefore, it is reasonable to suppose that our results are valid for examinations performed without an endorectal coil not only in 3.0-T scanners but also in modern 1.5-T scanners.

Our qualitative assessment showed that interobserver agreement was excellent. In addition, all of the cases in which there was gross capsular involvement or ECE ≥ 1.0 mm were identified by both observers, which is extremely important because it can lead to changes in the treatment strategy and prognosis.

Our study has some limitations. First, we used a retrospective design, which makes the study more susceptible to biases, especially selection bias. In addition, the low number of cases of microscopic and macroscopic ECE limits the power of the results, creating a need for further studies in order to validate them. Furthermore, the scale for the qualitative assessment of the capsule on T2-weighted images was chosen arbitrarily, because the most recent versions of the PI-RADS do not include risk stratification for this parameter.

In summary, our results indicate that the extent of capsular contact on DWI is an independent predictor of ECE in prostate cancer. The extent of capsular contact as measured on an ADC map appears to be an accurate predictor of ECE. However, adding ADC values to the extent of capsular contact does not appear to significantly improve diagnostic accuracy for detecting microscopic ECE in prostate cancer.

## Figures and Tables

**Table 5:** Univariate and multivariate logistic regression analyses of the combination of clinical and imaging variables with ECE, for both observers.

Variable	Observer 1			Observer 2	
	Univariate analysis Odds ratio ± SD (95% CI) *P*-value	Multivariate analysis Odds ratio ± SD (95% CI) *P*-value		Univariate analysis Odds ratio ± SD (95% CI) *P*-value	Multivariate analysis Odds ratio ± SD (95% CI) *P*-value
Serum PSA	1.08 ± 0.04 (1,01–1.16) 0.02	0.93 ± 0.97 (0.09–1.91) 0.42		1.08 ± 0.04 (1.01–1.16) 0.02	1.06 ± 0.52 (0.97–1.17) 0.19
Gleason score (ISUP grade)	2.59 ± 0.75 (1.46–4.58) 0.001	1.95 ± 1.08 (0.66–5.77) 0.22		2.59 ± 0.75 (1.46–4.58) 0.001	1.66 ± 0.68 (0.74–3.71) 0.22
Positive fragments on biopsy (%)	1.02 ± 0.01 (1.00–1.05) 0.02	1.00 ± 0.02 (0.96–1.05) 0.71		1.02 ± 0.01 (1.00–1.05) 0.02	0.99 ± 0.02 (0.96–1.03) 0.68
Tumor size	1.19 ± 0.07 (1.06–1.33) 0.002	1.26 ± 0.18 (0.94–1.68) 0.11		1.16 ± 0.07(1.02–1.31) 0.02	1.12 ± 0.15 (0.87–1.47) 0.38
PI-RADS	3.65 ± 1.90 (1.31–10.10) 0.01	2.09 ± 1.62 (0.45–9.55) 0.34		4.80 ± 2.91 (1.47–15.60) 0.009	0.37 ± 0.48 (0.02–4.79) 0.44
CCT2	1.08 ± 0.03 (1.01–1.15) 0.02	1.07 ± 0.11 (0.87–1.32) 0.48		1.17 ± 0.05 (1.07–1.28) 0.0001	0.92 ± 0.10 (0.73–1.17) 0.53
CCDWI	1.06 ± 0.32 (1.00–1.12) 0.04	0.92 ± 0.10 (0.74–1.14) 0.46		1.14 ± 0.04 (1.06–1.23) 0.0001	1.10 ± 0.11 (0.90–1.34) 0.35
Mean ADC	6.58 ± 11.70 (0.20–214) 0.23	0.07 ± 0.81 (0.01–16.2) 0.34		783.2 ± 1890.5 (6.90–8879) 0.006	955.4 ± 3643.4 (0.54–1682) 0.07

SD, standard deviation; 95% CI, 95% confidence interval.
